# Prevalence and progression of erosive tooth wear among children and adolescents in a Swedish county, as diagnosed by general practitioners during routine dental practice

**DOI:** 10.1016/j.heliyon.2021.e07977

**Published:** 2021-09-11

**Authors:** Agneta Hasselkvist, Kristina Arnrup

**Affiliations:** a Dental Research Department, Public Dental Service, Region Örebro County, Örebro, Sweden; b University Health Care Research Center, Faculty of Medicine and Health, Örebro University, Örebro, Sweden

**Keywords:** Tooth erosion, Children and adolescents, Prevalence, Progression, Dental general practices

## Abstract

**Objectives:**

To investigate the prevalence and four-year progression of erosive tooth wear (ETW) recorded in general dental practice, and to evaluate the usefulness of a simplified grading scale.

**Methods:**

Four cohorts (aged 3, 7, 11 and 15 years at baseline; n = 735) were followed from 2008 to 2012 during their routine dental examinations. Grading of ETW was performed on permanent upper incisors and first molars, using the scales of Johansson et al. 1996 and Hasselkvist & Johansson 2010**.**

**Results:**

Valid data were available for 641 individuals, 7-19-years of age, of whom 326 had data allowing analyses of progression. The prevalence of ETW increased with age, although at a lower level than in comparable studies. Progression was found in one-third of the subjects, with higher proportions and higher grades noted among the older cohorts. The simplified scale, that graded only four surfaces, resulted in just a few missed, mainly mild, cases of ETW.

**Conclusions:**

Clinically significant signs of ETW and patterns of progression can be reliably detected if the erosion index used includes a few selected surfaces of permanent teeth as part of the routine dental examination. Early signs of ETW, however, seem to be more difficult to detect and evaluate.

**Clinical significance:**

It is both possible and beneficial to introduce the diagnosing of ETW in routine dental examinations. To reduce the time involved in grading every patient, the simplified 4-surface application, seems to be a useful tool, but which is to be augmented with more extensive grading in individuals considered to be at risk.

## 1 Introduction

Epidemiological studies have reported a high prevalence of erosive tooth wear (ETW) among children and adolescents over the past few decades. A recent narrative review found prevalence of ETW varying between 0 and 100% in countries all over the world, while also showing a wide range in severity [1]. In primary teeth, some studies included in said review reported that more than 80% of erosion cases were confined to enamel, while other studies showed 21–48% of cases had dentine involvement. In permanent teeth, prevalence of specified erosive lesions into dentine ranged between 2-30%, and a wide variation was reported even in studies within the same country. Another review and meta-regression analysis found that the overall estimated prevalence of ETW was 30.4% in 12–19 year-old children and adolescents [2], although lesions in enamel and lesions into dentine were not specified. In addition, several of the included studies in the review did not discriminate between ETW and tooth wear. Studies on adults are scarce, but a recent Swedish study in a population of 20–89 year-olds found almost 80% showing signs of ETW [3].

Such wide variations in prevalence may be variously explained: studies are performed in different parts of the world, among different samples, sex and age cohorts, and the environments at the investigation sites differ (viz. studies are commonly performed in schools or in dental clinics), etc. Furthermore, one or multiple investigators may be involved in the recording process, a variety of scales may be used for grading, and the results may be based on different outcome measures. All these differing circumstances mean that the results of different studies may be difficult to compare. Nevertheless, a common feature of the studies is that the grading of the teeth would have been preceded by a period of education and training of the investigators in the grading system to be applied [1].

There have been three Swedish studies published on ETW among 17-20 year-olds, all showing similar results: 18–22% show severe lesions (into dentine) in at least one tooth [4, 5, 6]. The results of these studies should be reasonably comparable, since the grading of ETW in these studies were performed by trained investigators using the same scale for grading erosive lesions [4, 7], as well as the same choice of outcome measure. Norwegian studies have shown similar results, with 14–15% of 16–18 year-olds [8] and 20% of 16 year-olds [9] having lesions into dentine (using another scale, VEDE, Visual Erosion Dental Examination system, for grading erosive lesions).

Because of the high prevalence of ETW among adolescents, it would seem important that regular oral health examinations should incorporate an identification and diagnosis of dental erosion so as to facilitate taking adequate measures when erosive lesions or severe risk for ETW are detected. The accuracy of grading ETW in routine clinical practice by examiners without special training is, however, unclear.

The primary aim of this study was to investigate the outcome in terms of the prevalence of ETW, of grading of ETW performed by several investigators in routine general dental practice settings. Additional aims were to study incidence and progression of ETW over four years and to investigate any differences in prevalence, incidence and progression by cohort/age and gender. Finally, the study aimed to evaluate the usefulness of a simplified application of a grading system that grades only 4 instead of 12 surfaces.

## 2 Materials and methods

### 2.1 Study population

Between 2008-2012 the BITA study (Barn I TAndvården = Children in dental care) took place in five Public Dental clinics in Region Örebro County (ROC) and eight Public Dental Clinics in the Region of Västra Götaland (RVG). The clinics represented both rural and urban societies of different sizes and social demography. The BITA study dealt with different aspects of dental care in children and adolescents and followed four age cohorts (3-, 7-, 11- and 15-year-olds at the start of the study) over four years. Grading of dental erosion was included in the ROC part of the study.

Of 3134 patients in both regions, 2363 (75%) accepted to participate in the BITA study, 1628 in RVG (without ETW measurements included) and, comprising the study population for this report, 735 in ROC. The participants in the four age cohorts were examined at baseline (2008) and scheduled for reexamination in at least 2010 and 2012. Erosion data from 2008 and 2012 were used, but in case of missing data from 2008, it was replaced by data, if available, from 2009 (n = 10) or 2010 (n = 2). Missing data from 2012 were replaced by data from 2011 (n = 2) to enable maximal use of available data (Table 1). Consequently, ages may vary by between 1-2 years in a few individuals in the respective age cohort.

**Table 1:** Sample characteristics of individuals included in the Region Örebro County (ROC) part of the BITA study; number of individuals with valid Erosive Tooth Wear (ETW) registrations on permanent teeth at baseline and/or follow up by cohort/age and gender.

Cohort	Birth year	Gender	Included in the ROC part of the BITA study, n	Time point 2008 Baseline ETW registrations		Time point 2012 Follow-up ETW registrations		ETW registrations at both time points
				Age 2008	Valid n	Age 2012	Valid n	Valid n
1	2005	Female	120	3		7	79	
		Male	97	3		7	62	
		All	217	3		7	141	
2	2001	Female	114	7	73	11	98	62
		Male	113	7	72	11	98	61
		All	227	7	145	11	196	123
3	1997	Female	95	11	74	15	80	64
		Male	67	11	63	15	53	50
		All	162	11	137	15	133	114
4	1993	Female	69	15	61	19	55	49
		Male	60	15	55	19	44	40
		All	129	15	116	19	99	89
**Total**			**735**		**398**		**569**	**326**

### 2.2 Data collection

Data collection in the BITA study was performed by the dentists who were on duty at the five ROC clinics during the examination periods. Prior to the start of the study the examiners had received a concise (30 min) education, including a theoretical review of grading ETW and calibration on photographs. As a complement, all the examiners had access to a manual with written definitions and examples of the different scale steps on photographs. During the study period some of the dentists were replaced, and the new examiners had to rely mainly on the manual, though the project leaders visited the clinics at least yearly to remind the examiners of the routines of the study. Clinical data, including grading of ETW, were entered by the dental personnel into the MedView program [10], which is a computer system for formalized registration and subsequent analysis of clinical and image-based information.

### 2.3 Clinical examination

The regular clinical dental examination was supplemented with grading of ETW on buccal/palatal and incisal/occlusal surfaces on all four permanent upper incisor teeth and all four first permanent molars. The recording took place in a regular clinical setting with adequate operative lighting and ordinary mouth mirrors, using the scales according to Johansson et al 1996 for grading ETW on buccal and palatal surfaces on upper front teeth, and of Hasselkvist & Johansson 2010 for grading cupping on occlusal surfaces [4, 7] (Table 2).

**Table 2:** Ordinal scale used for grading severity of erosive tooth wear (ETW) by recording dental erosion on buccal and palatal surfaces of maxillary anterior teeth and cuppings on occlusal surfaces of first permanent molars.

Grade	Criteria	
	Anterior teeth	Molars
0 none	No visible changes, developmental structures remain, macro-morphology intact	No cupping/intact cusp tip
1 mild	Smoothened enamel, developmental structures have totally or partially vanished. Enamel surface is shiny, matt, irregular, “melted”, rounded or flat, macro-morphology generally intact	Rounded cusp tip
2 moderate	Enamel surface as described in grade 1. Macro-morphology clearly changed, facetting or concavity formation within the enamel, no dentinal exposure	Cupping ≤1 mm
3 severe	Enamel surface as described in grades 1 and 2. Macro-morphology greatly changed (close to dentinal exposure of large surfaces) or dentine surface exposed by at least 1/3	Cupping >1 mm
4 very severe	Enamel surface as described in grades 1, 2 and 3. Dentine surface exposed by at least 1/3 or pulp visible through the dentine	Fused cuppings: at least two cuppings are fused together on the same tooth

### 2.4 Outcome measures

Only permanent teeth were analyzed in this study. Buccal and palatal surfaces of upper incisors and occlusal surfaces of first molars were included, producing a maximum of 12 graded surfaces per individual [4, 7]. To determine the severity of ETW in one individual, peak erosion value (the highest score of erosion and/or cupping on any surface included in the index applied) was used as outcome measure.

In addition, a simplified application of the grading system, scoring only four surfaces (palatal surfaces on upper central incisors and occlusal surfaces of first lower permanent molars, i.e. the Simplified Erosion Partial Recording System (SEPRS) was applied and the outcome was presented as SEPRS peak erosion values [4].

### 2.5 Statistical analysis

The results are reported using descriptive statistics in terms of frequency distributions and cross-tabulations. Differences between cohorts/ages and genders, in prevalence at the two time points and in 4-year progression, were analyzed using logistic regression (ENTER) models. As dependent variable in the analyses of prevalence, a dichotomization with the cut-off at peak erosion value of ≥1 was used. For the analysis of progression, the dependent variable was a peak erosion value change of at least one step. For evaluation of the usefulness of the simplified version of the grading system, sensitivity and miss rates were computed. The statistical analyses were performed in IBM SPSS statistics 25, 2017, and *p* values below 0.05 represented statistical significance.

## 3 Ethical approval

An application for ethical approval (No. 286-07) was submitted prior to the BITA study. The Ethics Review Committee found the project not to be subject to the Swedish Act on Ethical Review but commented on the information given to the patients, which was taken into account during further planning of the study. Parents and children (from 7 years of age) gave their written consent to participate in the study.

## 4 Results

Of the 735 individuals in ROC there were valid data on ETW for 641 individuals in the age range 7–19 years with at least one grading set on at least one occasion. The total number of valid registrations was 967, viz, 398 at baseline, and 569 at follow-up. In cohorts 2, 3 and 4, 326 individuals had ETW recorded at both baseline and at follow-up (Table 1).

The frequency distributions of ETW prevalence and severity (peak erosion values) were reported separately for baseline and follow-up, and by cohort/age and gender (Table 3).

**Table 3:** Prevalence and severity of Erosive Tooth Wear (ETW) as frequency distributions of individual peak erosion values (based on scoring of 12 surfaces) by time point, cohort/age and gender as well as in totals by time point. The frequency distributions of individual peak erosion values based on scoring of 4 surfaces (Simplified Erosion Partial Recording System, SEPRS) are given as totals for the respective time point.

Time point	Cohort	Birth year	Age at time point	Gender	Valid n	Frequency distribution of individual peak erosion values %				
						0	1	2	3	4
2008 (Baseline)	2	2001	7	Female	73	98.6	0	1.4	0	0
				Male	72	98.6	1.4	0	0	0
				Total	145	98.6	0.7	0.7	0	0
	3	1997	11	Female	74	81.1	16.2	2.7	0	0
				Male	63	81.0	17.5	1.6	0	0
				Total	137	81.0	16.8	2.2	0	0
	4	1993	15	Female	61	60.7	23.0	16.4	0	0
				Male	55	45.5	36.4	14.5	1.8	1.8
				Total	116	53.4	29.3	15.5	0.9	0.9
	**Total**	**12 surfaces**			**398**	**79.4**	**14.6**	**5.5**	**0.3**	**0.3**
		SEPRS 4 surfaces			390	80.3	14.1	5.1	0.3	0.3
2012 (Follow-up)	1	2005	7	Female	79	98.7	1.3	0	0	0
				Male	62	100	0	0	0	0
				Total	141	99.3	0.7	0	0	0
	2	2001	11	Female	98	88.8	8.2	1.0	2.0	0
				Male	98	87.8	10.2	2.0	0	0
				Total	196	88.3	9.2	1.5	1.0	0
	3	1997	15	Female	80	53.8	30.0	15.0	1.3	0
				Male	53	43.4	30.2	24.5	1.9	0
				Total	133	49.6	30.1	18.8	1.5	0
	4	1993	19	Female	55	36.4	34.5	20.0	9.1	0
				Male	44	36.4	29.5	15.9	15.9	2.3
				Total	99	36.4	32.3	18.2	12.1	1.0
	**Total**	**12 surfaces**			**569**	**72.9**	**16.0**	**8.1**	**2.8**	**0.2**
		SEPRS 4 surfaces			563	74.1	15.5	7.8	2.7	0

The overall prevalence of visible ETW (peak erosion value ≥ 1) was 20.6% at baseline (cohorts 2–4: ages 7, 11 and 15, respectively) and 27.1% at follow-up (cohorts 1–4: ages 7, 11, 15 and 19, respectively). At age 7 (cohort 2 at baseline and cohort 1 at follow-up), close to 100% showed no signs of ETW (peak erosion value 0) and equally, no severe cases (peak erosion value 3 or 4) were detected. Higher prevalence of ETW was seen at older ages both at baseline and at follow-up (Table 3) and a few severe cases were identified. At age 19 (cohort 4 at follow-up), the prevalence of ETW was 63.6% while 13.1% were identified as severe cases.

The 4-year progression of ETW was reported by cross-tabulating the peak erosion values per individual at baseline and at follow-up by cohort for the 326 individuals who had data that were comparable. Of these 326 individuals, 25 were graded with higher values at baseline than at follow-up and who were subsequently graded as improved (viz. 2, 9, and 14 individuals in cohorts 2, 3, and 4, respectively). These were counted as unchanged in the analyses. In total, 107/326 individuals (32.8%) were graded as deteriorated by at least one scale step (viz. 13, 51 and 43 cases in cohorts 2, 3 and 4, respectively). In cohort 2, 10.6% showed progression of ETW, while even higher proportions in the older cohorts showed progression, viz. 44.7% in cohort 3 and 48.3% in cohort 4. The older cohorts also progressed on to higher grades of ETW (Table 4).

**Table 4:** Changes (figures in bold) in individual peak erosion values from baseline to follow-up for the 326 participants with valid registrations at both time points illustrated by cross-tabulation of the frequency distributions. Deterioration is reported as percentage of individuals with an increase of at least one scale step.

Cohort	Birth year	Valid n	Grading at baseline	Grading at follow-up					Deterioration %
				0	1	2	3	4	
2	2001	123	0	109	**11**	**1**	**1**		10.6
			1		1				
3	1997	114	0	48	**27**	**17**	**1**		44.7
			1		13	**5**	**1**		
			2			2			
4	1993	89	0	19	**20**	**5**	**7**		48.3
			1		18	**5**	**1**		
			2			8	**4**		
			3					**1**	
			4					1	
**All**		**326**							**32.8**

A majority of those who showed progression (90/107) was graded at baseline with peak erosion value 0, i.e. did not show any signs of ETW (Table 4). Of the 326 individuals included in the analyses of progression, 266 showed no signs of ETW at outset (peak erosion value 0), giving a 4-year incidence rate of 90/266 = 33.8% (Table 5).

**Table 5:** Differences in prevalence and progression of Erosive Tooth Wear (ETW) at or between the two time points by cohort/age and by gender, analysed using logistic regression models (ENTER) for valid cases. As dependent variable in the analyses of prevalence, a dichotomization with the cut-off at peak erosion value of ≥1 was used. For the analysis of progression, the dependent variable was a peak erosion value change of at least one step, and the model was adjusted for ETW at baseline. Odds Ratios, 95% Confidence Intervals and *p*-values are given in the table.

Prevalence at time point 2008 (Baseline); n = 398; ETW peak value ≥ 1, yes = 1 (n = 82; 20.6%), no = 0 (n = 316)							
Independent variable		Valid n	Proportion peak erosion values ≥ 1		OR	95% CI	*p*-value
			Count	%			
Cohort	Birth year						<0.001
2	2001	145	2	1.4	0.016	0.004–0.067	<0.001
3	1997	137	26	19.0	0.268	0.153–0.417	<0.001
4 (ref)	1993	116	54	46.6			
Gender	Female (ref)	208	39	18.8			
	Male	190	43	22.6	1.41	0.817–2.436	0.217

Prevalence at time point 2012 (Follow-up); n = 569; ETW peak value ≥ 1, yes = 1 (n = 154; 27.1%), no = 0 (n = 415)							
Independent variable		Valid n	Proportion peak erosion values ≥ 1		OR	95% CI	*p***-value**
			Count	%			
Cohort	Birth year						<0.001
1	2005	141	1	0.7	0.004	0.001–0.030	<0.001
2	2001	196	23	11.7	0.075	0.041–0.136	<0.001
3	1997	133	67	50.4	0.584	0.343–0.995	0.048
4 (ref)	1993	99	63	63.6			
Gender	Female (ref)	312	84	26.9			
	Male	257	70	27.2	1.194	0.761–1.876	0.440

Progression between the two time points (Baseline to Follow-up); n = 326; ETW peak erosion value change ≥1, yes = 1 (n = 107; 32.8%), no = 0 (n = 219)							
Independent variable		Valid n	Proportion peak erosion value change ≥1		OR	95% CI	*p***-value**
			Count	%			
Cohort	Birth year						<0.001
2	2001	123	13	10.6	0.074	0.034–0.162	<0.001
3	1997	114	51	44.7	0.635	0.347–1.164	0.142
4 (ref)	1993	89	43	48.3			
Gender	Female (ref)	175	56	32.0			
	Male	151	51	33.8	1.342	0.799–2.254	0.267
ETW at baseline (peak erosion value ≥ 1)							
	No (ref)	266	90	33.8			
	Yes	60	17	28.3	0.292	0.146–0.584	0.001

### 4.1 Logistic regression analyses

For evaluation of differences in prevalence or progression by cohort/age and by gender, logistic regression analyses were performed. As the dependent variable in the analyses of prevalence, a dichotomization with the cut-off at peak erosion value of ≥1 was used (Table 5). Using the oldest cohort (4: age 15 in 2008 and 19 in 2012) as the reference category, the prevalence in all younger cohorts were significantly lower at both time points (OR from 0.004 for the youngest to 0.584 for cohort 3, age 15 at follow-up; Table 5). Differences by gender were not significant (Table 5).

In the analysis of differences in progression, the dependent variable was set as a peak erosion value change of at least one step between baseline and follow-up. Differences in progression were significant by cohorts/ages but not by gender (Table 5). Using the oldest cohort (4: from age 15 to age 19 over the 4-year-progression period) as reference category in the logistic regression analyses, the progression was significantly lower (OR 0.074; Table 5) in the youngest cohort (2: from age 7 to age 11 over the period), while the OR 0.635 for progression in cohort 3 (from age 11 to age 15), was non-significant (Table 5).

### 4.2 Usefulness of the simplified grading application

Using the simplified application of the grading system (SEPRS), the number of valid registrations decreased to 390 and 563, respectively and the overall prevalence of ETW were 19.7% at baseline and 25.9% at follow-up (Table 3). Based on the dichotomization of peak erosion value ≥ 1, sensitivity of the SEPRS version, with the outcome that graded 12 surfaces considered as the true value, was 93.9% for the baseline assessments and 97.3% for the follow-up data. Miss rates were thus 6.1% (5/82 cases) and 2.7% (4/150 cases), respectively. Two of the missed cases at follow-up were graded as severe using the 12-surface version.

## 5 Discussion

In the BITA study the grading of ETW was performed in settings akin to routine general dental practice, by examiners with limited training or calibration prior to the examinations. This makes it interesting to compare our results of ETW with those of other studies. Overall, there was broad concordance with other studies as regards the pattern of age-related increase in prevalence and severity of ETW, although at a lower level. For gender differences, the BITA study revealed no significance.

In the two younger cohorts (ages 7- and 11-years), the prevalence of visible ETW was low and since the prevalence of ETW in permanent teeth in other studies in children and adolescents vary from 0 to 100% [1], it may well reflect the reality of wide variability as regards prevalence. The low severity of ETW in these youngest age cohorts with newly erupted teeth would be reasonable to expect. Severe ETW (grades 3 or 4, which represents tooth loss into dentine) was registered in none of the 7 year-olds and just a few of the 11 year-olds, which is again similar to studies on similar ages in Iceland and in the Netherlands [11, 12, 13].

Although both prevalence and severity of ETW became generally more frequent with older age, which is in line with other studies [1, 4, 6, 11], a lower prevalence and severity than in comparable studies was found for our older cohorts. Both at age 15 years and at age 19 years, less severe ETW compared to the other Swedish studies was found [4, 5, 6]. This picture of lower prevalence and severity figures suggests that ETW may have been understated under the circumstances of a setting akin to routine general dental practice, as opposed to a controlled research setting.

In a European context, there are only a few previous studies on progression of ETW. Our results of about a third of the participants who had valid data for comparison of higher graded ETW at follow-up are well in line with another Swedish 4-year study of adolescents aged 13–14 years at baseline. On the other hand, incidence rates differ more widely between studies, with our 4-year incidence rate of 34% being within a range of between 12% in a British study following 12-year-olds over 2 years, 27% in a 3-year longitudinal Dutch study of children aged 10–12 years at baseline [14, 15] and 76% in the above-mentioned Swedish 4-year study [16]. Thus, the wide range in incidence rates in the studies may partly be due to different aged cohorts and different follow-up periods. However, our incidence results compare well with both the British and Dutch studies, taking the varying follow-up periods into account.

In line with the pattern of age-related increases in prevalence and severity, the significantly lower progression of ETW in cohort 2, compared with cohorts 3 and 4, was an expected finding. The rapid increase in prevalence and severity during adolescence points to the importance of paying full attention to early signs of ETW in childhood and early adolescence. By regularly recording lower grades it should be easier to detect progression so as to prevent serious tissue loss.

In the BITA study no gender difference could be detected, neither in prevalence, severity, progression nor incidence of ETW. There are contradictory results even within the Swedish studies, with males showing higher prevalence and severity than females in two studies [4, 6] while in a study on 20 year-olds no gender difference was reported [5].

An important explanation for the fact that the main part of progression recorded were changes from no signs of ETW to any signs of ETW could be the construction of the scale applied. The first step in the scale (from 0 to 1) describes a small loss of surface characteristics and subsequently a minor deterioration may be visually registered (but could conceivably also be missed), while changes between the higher grades are based upon successively greater amounts of surface loss between each scale step. Thus, the misdetection of changes indicating some progression, although not covered by the scale, on surfaces that had at baseline already been graded with any sign of ETW, cannot be excluded.

The choice of the scales for the recording of ETW was based on previous experience of these scales in another study within the district [4], and this also facilitates comparison. The 5-graded scale allows the grading of early signs of ETW with time since there are two steps before dentine involvement. The use of visual grading of ETW according to specified scales may be somewhat primitive and even arguably somewhat insecure, and the intra- and inter-examiner reliability (which is by definition not easy to measure in a large group of examiners) can be supposed to vary considerably. More sophisticated instruments, such as optical reflectometer, are still under development but are not yet available in general practice [17]. Another method that has been shown to be useful for diagnosing ETW is to perform the grading on 3D images [18]. These kinds of more accurate instruments will eventually become available, but presently, and in general practice, one has to rely on visible grading.

Dental care in Sweden is free of charge for children and adolescents and is open to individual choice between private or public care. The vast majority of these age groups are, by their own choice, listed at clinics within the system of Public Dental Care. The five Public Dental Clinics in this study were selected to represent societies of different sizes and socio-demographics. The examiners were general practitioners employed in these Public Dental Clinics. Some examiners were involved during the entire data collection period, while some took part for shorter periods. At the same time, it is quite common that there is a considerable mobility of employees, the setting may be considered representative of Swedish Public Dental Care in general. The BITA project aimed to study several aspects of dental care of children. The design with four age cohorts followed over 4 years, aimed to allow observations over the age-span 3–19 years. The size of the cohorts was calculated for the main purpose of the study, but the 735 included individuals may be considered large enough for the erosion part of the study.

At the start in 2008, most dental professionals were rather unfamiliar with the grading of ETW, when it was added as a new, but not a main, part of the oral health examination within the study. Thus, the grading of ETW was performed as an ‘add-on’ aspect of the clinical examination and data collection, and not as the main focus as would be the case in a study focused on erosion, which usually would have been the case in other studies on ETW. From the perspective of seeking the true prevalence, this must be considered as a limitation. On the other hand, it might also be considered a strength when viewed with respect to the usefulness of the applied diagnostic tools (visual grading, scales and indexes) for routine general dental practice.

In clinical practice it would be advantageous to reduce the time consumed on examining and diagnosing healthy individuals. In this regard, the use of SEPRS that involves grading of only four surfaces proved to work out well, with only a few undetected, nearly exclusively mild, cases of ETW noted. The tooth surfaces used in this scale have previously been clearly shown to be the surfaces most affected by ETW [11, 12, 13].

One reason to introduce grading of ETW in general practice should be to detect, diagnose and take adequate measures to prevent deterioration. It is important to identify patients at risk for developing severe tooth surface loss, enabling preventive measures to be taken and, if needed, to perform adequate treatment in time. In spite of the short preparation for the examiners prior to the study, peak erosion values of 3 or 4 was found in 13% of 19-year-olds, indicating that severe ETW may well be diagnosed with some accuracy in routine general practice. The important grading of early signs of ETW was, perhaps unsurprisingly, less promising, which may be due both to lack of education and time.

The increasing knowledge about and attention to ETW, combined with training in grading and diagnosis may probably already have increased the ability of the examiners involved here to detect and follow-up both early and more severe signs, take preventive measures and offer adequate therapy. Still, a main purpose must be to identify individuals at risk before developing more severe tooth damage. In the Swedish context, at the time of writing, a national system is being implemented for grading and detecting patients at risk. However, there is still a lack of general guidelines adapted to age for managing patients at risk for progression of ETW.

## 6 Conclusions and implications

It is possible to diagnose severe ETW on selected permanent teeth with some accuracy in general dental practice, if minor education has been provided. However, prevalence was lower than expected and the risk of missing less severe conditions cannot be discounted. The progression of ETW shows the same pattern as in other studies, albeit at a lower level.

More education and training should probably improve the skills among the examiners to detect individuals both in need of taking measures for already existing ETW and for individuals at risk for deterioration of less severe conditions. The latter implies a need of useful tools for screening and higher skill of examiners to detect, monitor and evaluate lower grades of ETW.

To reduce the time consumed for grading every patient, the 4-surface scale, SEPRS, seems to be a useful tool, to be followed by a more extensive grading on individuals at risk.

## Declarations

### Author contribution statement

Kristina Arnrup: Conceived and designed the experiments; Performed the experiments; Analyzed and interpreted the data; Contributed reagents, materials, analysis tools or data; Wrote the paper.

Agneta Hasselkvist: Analyzed and interpreted the data; Wrote the paper.

### Funding statement

This work was supported by the Public Dental Service, Region Örebro County.

### Data availability statement

Data will be made available on request.

### Declaration of interests statement

The authors declare no conflict of interest.

### Additional information

No additional information is available for this paper.
